# Diverse perspectives on proteomic posttranslational modifications to address EGFR-TKI resistance in non-small cell lung cancer

**DOI:** 10.3389/fcell.2024.1436033

**Published:** 2024-12-24

**Authors:** Yuhong Ma, Feng Zhang, Jin Li, Juan Li, Yanhua Li

**Affiliations:** ^1^ Department of Radiotherapy Oncology, The Second Affiliated Hospital of Dalian Medical University, Dalian, China; ^2^ Department of International Medical Department, The Second Affiliated Hospital of Dalian Medical University, Dalian, China

**Keywords:** EGFR-TKI resistance, phosphorylation, autophosphorylation, glycosylation, crosstalk, NSCLC

## Abstract

Non-small cell lung cancer (NSCLC) is the main histological subtype of lung cancer. For locally advanced and advanced NSCLC, epidermal growth factor receptor (EGFR) tyrosine kinase inhibitor (TKI)-targeted therapy has been the first choice for NSCLC patients with EGFR mutations. TKIs, as targeted drugs, inhibit kinase activity and autophosphorylation by competitively binding to the ATP binding site of the EGFR tyrosine kinase domain, which blocks the signal transduction mediated by EGFR and thus inhibits the proliferation of tumor cells. However, drug resistance to TKIs is inevitable. EGFR is also a highly glycosylated receptor tyrosine kinase, and a wide range of crosstalk occurs between phosphorylation and glycosylation. Therefore, can the phosphorylation state be altered by glycosylation to improve drug resistance? In this review, we summarize phosphorylation, glycosylation and the crosstalk between these processes as well as the current research status and methods. We also summarize the autophosphorylation and glycosylation sites of the EGFR protein and their crosstalk. By exploring the relationship between EGFR glycosylation and autophosphorylation in targeted TKI therapy, we find that research on EGFR glycosylation is crucial for targeted NSCLC treatment and will become a research direction for identifying potential targets related to regulating TKI drug sensitivity.

## 1 Introduction

Lung cancer is a malignant tumor with the highest morbidity and mortality of all cancers worldwide (Sung et al., 2021). NSCLC is the main histological subtype of lung cancer, accounting for more than 85% of all lung cancer cases (Wang et al., 2021). Lung cancer treatment includes traditional surgery, radiotherapy, chemotherapy or a combination of these treatments; however, the survival rates and overall cure rates remain low (Herbst et al., 2018). In recent years, targeted EGFR-TKI therapy has been the first choice for patients with advanced NSCLC and EGFR mutations, which has improved the quality of life of these patients.

Mechanistically, in non-small cell lung cancer, mutations in EGFR lead to destabilization of the nonactive conformation of the EGFR protein and continuous activation of its kinase domain (Di Maio, 2023), which leads to continuous autophosphorylation of the C-terminal tyrosine site and results in tumorigenesis (Irmer et al., 2007; Reita et al., 2021). In contrast, TKIs inhibit kinase activity and autophosphorylation by competitively binding the ATP binding site of the EGFR tyrosine kinase domain and blocking EGFR-mediated signal transduction, thus inhibiting tumor cell proliferation (Pao and Chmielecki, 2010; Roskoski, 2014). However, drug resistance is inevitable (Cooper et al., 2022; Noronha et al., 2022).

In addition, EGFR is a highly glycosylated receptor tyrosine kinase (Han et al., 2021; Xiao et al., 2023). Many studies have confirmed extensive crosstalk between glycosylation and phosphorylation (Patwardhan et al., 2021; Pienkowski et al., 2023). Therefore, the study of proteomic posttranslational modifications (PTMs) of EGFR can provide a basis for the discovery of potential glycosylation-related targets in EGFR-TKI therapy for NSCLC.

## 2 Phosphorylation and glycosylation PTMs in proteins

The central dogma of molecular biology is that genetic information in a biological system is transferred from DNA to RNA and then to protein. However, butterflies and caterpillars share the same genome but have different morphologies, which is attributed mainly to PTMs (Crick, 1970). After RNA is translated into a protein, a covalent chemical modification of specific amino acid residues occurs, which is called a protein PTM. PTMs are ubiquitous in organisms and contribute to almost all cellular activities, especially signal transduction. In some cases, PTMs can act as molecular switching valves to activate or inhibit signaling pathways and to change the life processes of proteins (Zecha et al., 2023). PTMs endow proteins with additional forms of expression, which increases the complexity of human life processes (Keenan et al., 2021; Geffen et al., 2023).

To date, nearly 500 types of protein PTMs have been reported (Keenan et al., 2021), including ubiquitination, methylation, acetylation, glycosylation, and phosphorylation (Zhu et al., 2021). Different PTMs not only affect protein folding and conformational stability but also play important roles in cell development and transformation and the cell signaling network (Thygesen et al., 2018) and serve as targets for the diagnosis and treatment of many types of tumors (Kuntz et al., 2017; Yang et al., 2018). Currently, the most studied and widely applied protein PTMs are phosphorylation and glycosylation, as these account for more than 50% of PTMs (Pagel et al., 2015). Each individual protein may contain multiple PTM sites, one of which can affect the removal or addition of another modification at the same proximal or distal location. This phenomenon is called PTM “crosstalk” (Vu et al., 2018; Cutler et al., 2021). For example, the crosstalk between phosphorylation and O-acetylglucosamine modification (O-GlcNAcylation) can be used as a nutrient/pressure sensor to regulate functions such as signal transduction, transcription and the cytoskeleton (Tang et al., 2023).

### 2.1 Phosphorylation

Phosphorylation, which is one of the most common PTMs of proteins, occurs on more than 30% of proteins in cells and is the most common, basic and important mechanism for regulating and controlling protein function (Cohen, 2002; Yadav et al., 2017). Phosphorylation is involved in the regulation of a variety of biological processes, and the overexpression of kinases and abnormalities in their regulatory mechanism can lead to the activation or imbalance of kinase signaling pathways, which is the basis of most cases of cancer development (Fujimoto et al., 2022; Li et al., 2023). During phosphorylation, under kinase catalysis, the γ phosphate group of ATP is transferred to the side chain hydroxyl group of threonine (T), serine (S) or tyrosine (Y) to form a phosphate ester. This process is reversible; that is, the phosphorylated group can be hydrolyzed and removed by phosphatase, which results in dephosphorylation (Hu et al., 2023). Phosphorylation and dephosphorylation are common regulatory modes that regulate protein conformation, protein cell localization and interactions between signaling molecules, and these modes are involved in cellular activities such as cell proliferation, migration, differentiation and apoptosis. Both phosphorylation and dephosphorylation have broad functions in signaling pathway transduction, apoptosis, development and differentiation, and cancer mechanisms, among other processes (Wang et al., 2022).

#### 2.1.1 Phosphorylation and disease

Many biochemical studies have focused on the treatment of diseases, such as autoimmunity (Aso et al., 2023), inflammation (Tu et al., 2022), metabolism (Batista et al., 2020), and degenerative diseases (Zheng et al., 2020), by interfering with phosphorylation-based signal transduction. For example, researchers combined global proteomics and phosphoproteomics data and revealed that diabetes leads to β-cell failure through a GSK3-PDX1-dependent axis, which aids in identifying potential islet-related therapeutic targets for human type 2 diabetes (Sacco et al., 2019). Suhas Vasaikar et al. used phosphoproteomics to analyze colorectal cancer and adjacent tissues and reported that Rb phosphorylation is an oncogenic driver in colorectal cancer. Targeting Rb phosphorylation in colorectal cancer via CDK2 inhibition represents a unique opportunity (Vasaikar et al., 2019). Furthermore, some studies have revealed a significant accumulation of phosphorylated tau in GABAergic interneurons of the dentate gyrus in Alzheimer’s disease patients, which subsequently inhibits GABAergic transmission and disinhibits the neural circuitry within the neurogenic niche, thereby impairing adult hippocampal neurogenesis and leading to cognitive decline in Alzheimer’s disease (Zheng et al., 2020). Additionally, researchers have used quantitative phosphoproteomics to reveal that tyrosine phosphorylation at key sites in the phosphatase SHP2 is inhibited and reduced, leading to subsequent suppression of the RAS/MAPK pathway and activation of the PI3K/AKT pathway. By performing inverse correlation analysis on a published drug-induced P100 phosphoproteomics dataset, they predicted that the combination of the PI3K/MTOR inhibitor dactolisib with osimertinib could overcome resistance to EGFR TKIs (Zhang et al., 2021). Therefore, understanding and intervening in the role of protein phosphorylation in tumors has become an important direction in cancer research.

### 2.2 Glycosylation

Glycosylation is a key posttranslational modification of proteins and encompasses the glycosidic bonds produced by enzymes between sugars and other sugars, proteins or lipids (Moremen et al., 2012). N-glycosylation and O-glycosylation are the two most common types of protein glycosylation (Bennett et al., 2012). Oligosaccharides involved in N-glycosylation are connected to asparagine residues and have a conserved pentasaccharide core structure, whereas oligosaccharides involved in O-glycosylation are linked to serine or threonine residues and are catalyzed by different glycosyltransferases, one monosaccharide at a time (You et al., 2018). Sugar chains are diverse due to differences in monosaccharide composition, connections between monosaccharides, heterogeneity, branching structures, the presence of other substituents and connections to their aglycones (Savarino et al., 2023). Glycosylation plays important roles in transcriptional activity (Myers et al., 2016; Constable et al., 2017), epigenetic regulation (Wu et al., 2017), neuronal function (Yim et al., 2022), response to external stressors (Ma et al., 2021) and regulation of protein‒protein interactions (Tarbet et al., 2018). Many studies on human glycosylation defects and their associations with disease have shown that mammalian sugar chains contain a large amount of biological information (Hoffman et al., 2023). Identification of the biological function of each sugar chain and sugar chain-binding protein has made important contributions to the diagnosis and treatment of multiple diseases (Davighi et al., 2023; Liang et al., 2023).

#### 2.2.1 Glycosylation and disease

Sialylation is an important type of cell glycosylation. The sialylated sugar chain structure plays an important role in cell recognition, adhesion and signal transmission. When glycosyltransferase expression changes, sialylation is related to the occurrence and development of cancer (Rossi et al., 2022). Serum CA199 is a tumor-related marker that is used clinically and is mainly detected in patients with pancreatic cancer, colorectal cancer, gastric cancer or cholangiocarcinoma. However, the expression of α2,3-sialylate type 1 (SLeA) is primarily detected for clinical diagnosis and monitoring of treatment response (Izquierdo-Sanchez et al., 2022).

Fucosylation, which involves synthesis by a series of fucosyltransferases, is an unextensible modification that is usually divided into terminal fucosylation and core fucosylation (Fujita et al., 2021). The final step of systemic lupus erythematosus antigen biosynthesis includes α1,3- or α1,4-fucosylation. An increase in core fucosylation is closely related to the occurrence of liver cancer (Loong et al., 2021). The core fucosylation of α-fetoprotein is a recognized biomarker for the early detection of hepatocellular carcinoma that helps distinguish between chronic hepatitis and liver cirrhosis (Sanda et al., 2021). In cancers, core fucosylation of EGFR is associated with increased dimerization and phosphorylation, which leads to an increase in EGFR-mediated signaling related to tumor cell growth and malignancy (Zhang et al., 2020; Ament et al., 2023).

The increased expression of the GlcNAc-N- sugar chain is due to the increased activity of GnT-V, which is encoded by the mannoside acetylglucosaminyltransferase 5 (MGAT5) gene. MGAT5 expression is regulated by the insulin-like growth factor 1 signaling pathway (Salvi et al., 2022). O-(ligated)-N-acetylglucosamine (O-GlcNAc), which is involved in a unique glycosylation reaction, is usually transferred to the hydroxyl groups of serine and threonine residues of proteins (Fan et al., 2023). O-GlcNAc glycosylation is very sensitive to uridine diphosphate-N-acetyl glucosamine. Uridine diphosphate-N-acetyl glucosamine is the donor of O-GlcNAc glycosylation (Ruan et al., 2013) and the downstream metabolite of glucose; thus, O-GlcNAc glycosylation is often referred to as a cellular nutritional receptor (Bond and Hanover, 2013). Like protein phosphorylation, O-GlcNAc plays an important role in regulating cell signaling and energy production (Yang and Qian, 2017). Loss of O-GlcNAc glycosylation has been shown to lead to cell dysfunction and even cell death (O'Donnell et al., 2004), whereas O-GlcNAc dysfunction is associated with many diseases, including Alzheimer’s disease, diabetes and other chronic diseases (Banerjee et al., 2016; Ma et al., 2017). The mucin-type O-glycosylation component GalNAc-type O-glycan, also known as mucin-O-glycan, is common in most transmembrane and secretory glycoproteins. Its abnormal expression is closely related to the development of malignant tumors (Berois et al., 2013). Furthermore, some studies have shown that the expression of GalNAc transferase, which initiates mucin O-glycosylation, also changes during the occurrence and development of cancer (Gornes et al., 2009).

Notch glycosylation is unique; the Notch signaling pathway is crucial for cell fate decisions, and its imbalance can lead to a variety of human diseases. Glycosylation of the extracellular domain of Notch has been shown to regulate its activity. Notch ligands bind to the receptor in the extracellular domain and activate it by inducing conformational changes in Notch, which exposes multiple cleavage sites. The cleavage of these sites leads to the release of the Notch intracellular domain, which shifts to the nucleus and controls the transcriptional activation of recombination signal binding protein inhibitors. The extracellular domain of Notch is modified by different types of carbohydrates, including N-glycans and O-polysaccharides (Moloney et al., 2000). Glycosylation-dependent Notch signal regulation controls development, maintains the “dryness” of tumor cells, and mediates cancer metastasis (Takeuchi and Haltiwanger, 2014).

### 2.3 Crosstalk between phosphorylation and glycosylation

**FIGURE 1:**
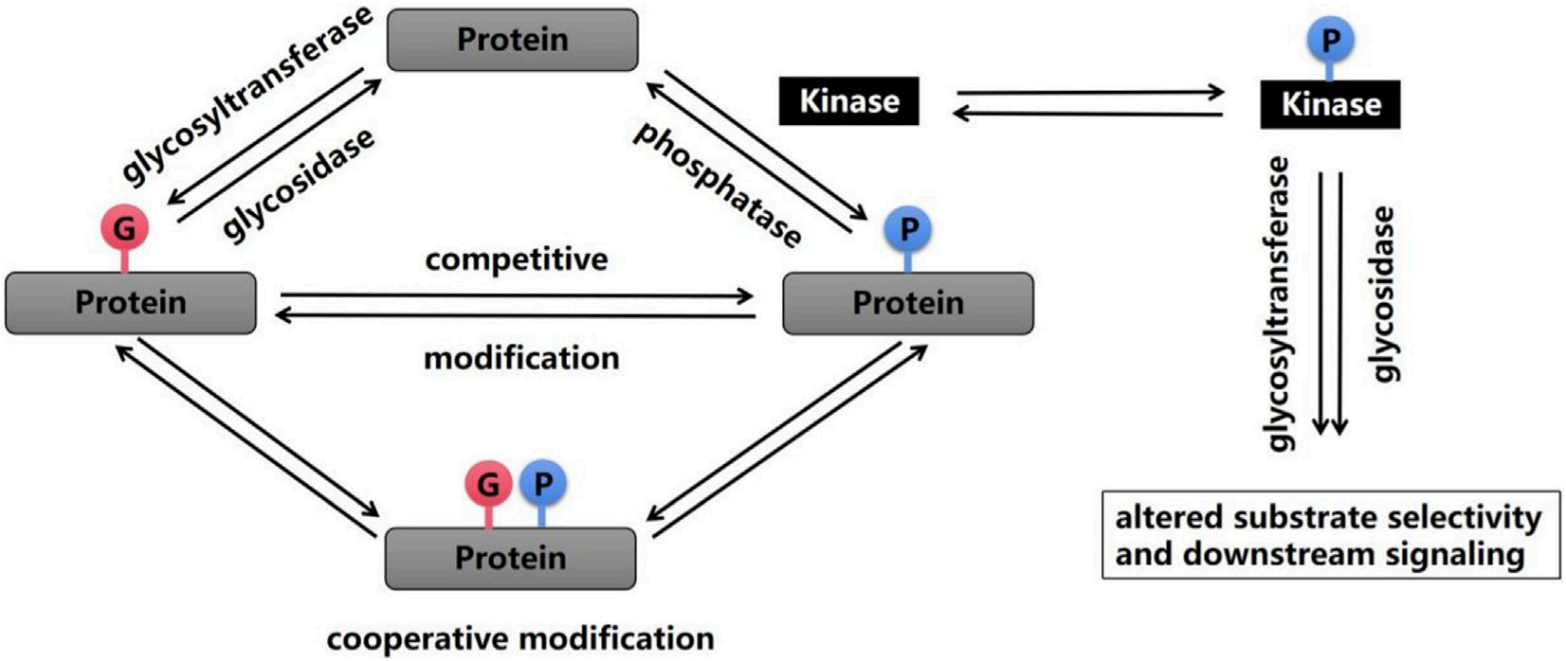
Phosphorylation and glycosylation are two important PTMs that are reversible by enzymatic action. A wide range of crosstalk between these two processes has been shown, and this crosstalk can occur on the same amino acid or at similar modification sites, which alters substrate selectivity and downstream signaling.

Most protein PTMs occur on specific amino acids, and the density of coexisting PTMs on proteins is very high; that is, a single protein may contain multiple different PTMs, and thus, the first modification may affect the addition or removal of the next modification (Schwein and Woo, 2020) (Figure 1). Multiple PTMs can positively or negatively affect other behaviors, which is called crosstalk between PTMs (Gan and Fan, 2024). An example of early evidence for the existence of crosstalk is that shortly after Hart et al. discovered O-GlcNAc glycosylation in 1984, researchers noticed that O-GlcNAc glycosylation occurred at known phosphorylation sites (Torres and Hart, 1984). The most studied form of PTM crosstalk is crosstalk between phosphorylation and O-GlcNAc glycosylation because these processes predominantly involve serine and threonine residues. When more than 800 phosphorylation sites were inhibited, the phosphorylation at 280 sites decreased, and the glycosylation at 148 sites increased. In addition, a change in O-GlcNAc glycosylation after the inhibition of serine/threonine phosphatases was also observed (Wang et al., 2008). Other studies have shown that calcium/calmodulin-dependent kinase IV (CaMKIV) is phosphorylated at multiple sites, including Thr200, and that CaMKIV undergoes O-GlcNAc glycosylation on at least five specific residues, including Ser137 and Ser189. The glycosylation of Ser137 leads to a slight increase in the phosphorylation of CaMKIV at Thr200, whereas the glycosylation of Ser189 leads to a substantial increase in phosphorylation at Thr200 (Dias et al., 2009). Consistent with these findings, the inhibition of GSK3β phosphorylation leads to an increase in O-GlcNAc glycosylation in COS7 cells (Wang et al., 2007). Currently, many cases of crosstalk between O-GlcNAc glycosylation and phosphorylation have been described, from which several individual modification sites have been identified, including Thr58 on c-Myc, which is modified by phosphorylation or O-GlcNAc glycosylation of the transcriptional activation domain (Kamemura et al., 2002), and the mouse estrogen receptor at Ser16, which regulates receptor activity (Chen et al., 2006).

## 3 Characteristics of glycosylation and autophosphorylation of EGFR in NSCLC TKI therapy

In recent years, targeted EGFR-TKI therapy has been the first choice for patients with advanced NSCLC and EGFR mutations; although the quality of life of patients has considerably improved with this treatment, drug resistance is inevitable. EGFR is currently an important target for targeted therapy of NSCLC; thus, the study of PTMs of EGFR can provide a basis for the discovery of potential glycosylation-related targets in EGFR-TKI therapy for NSCLC.

### 3.1 Characteristics of EGFR autophosphorylation

EGFR is a highly glycosylated tyrosine kinase type I transmembrane receptor of the ErbB family. Its total molecular weight is 170 kDa, and its protein sequence contains 1,186 amino acid residues, which constitute the N-terminal extracellular domain, transmembrane domain (TM), near-membrane domain (JM), tyrosine kinase domain (TK) and C-terminal domain (Chen et al., 2016; Amelia et al., 2022). EGFR binds to EGF and other ligands through the N-terminal extracellular domain to activate its conformation and dimerization; this binding also activates the intracellular tyrosine kinase domain, which causes the protein to self-phosphorylate its C-terminal tyrosine site and then activate downstream intracellular signaling pathways, such as the Ras/Raf/MEK/ERK, PI3K/AKT (PKB) and PLC γ pathways. This initiation includes a series of cellular physiological and pathological effects, such as cell proliferation, differentiation, apoptosis, angiogenesis, tumor invasion and metastasis (Ferguson, 2008; Seshacharyulu et al., 2012). At present, many confirmed mutations in EGFR phosphorylation sites cause disease (Table 1).

**TABLE 1:** Proven EGFR mutation sites associated with disease-related phosphorylation.

Positions	Sequence status	Mutagenesis sequence	Description	Publications
275	271-280: NPTTYQMDVN	Y → A	Strongly reduces autophosphorylation and activation of downstream kinases when associated with A-309	Ogiso et al. (2002)
287	281-290: PEGKYSFGAT	F → A	Strongly reduces autophosphorylation and activation of downstream kinases when associated with A-309	Ogiso et al. (2002)
309	301-310: VTDHGSCVRA	R → S	Strongly reduces autophosphorylation and activation of downstream kinases when associated with A-275/A-287	Ogiso et al. (2002)
429	421-430: ENLEIIRGRT	R → E	Abolishes autophosphorylation and activation of downstream kinases	Ogiso et al. (2002)
688	681-690: RLLQERELVE	L → A	Strongly reduces phosphorylation	Brewer et al. (2009), Jura et al. (2009)
689	681-690: RLLQERELVE	V → A	Reduces autophosphorylation	Brewer et al. (2009)
690	681-690: RLLQERELVE	E → A	Reduces phosphorylation	Brewer et al. (2009), Jura et al. (2009)
692	691-700: PLTPSGEAPN	L → A or P	Strongly reduces phosphorylation	Brewer et al. (2009), Jura et al. (2009)
693	691-700: PLTPSGEAPN	T → A	Increases phosphorylation	Brewer et al. (2009)
693	691-700: PLTPSGEAPN	T → D	Strongly reduces phosphorylation	Brewer et al. (2009)
694	691-700: PLTPSGEAPN	P → A	Strongly reduces phosphorylation	Brewer et al. (2009)
699	691-700: PLTPSGEAPN	P → A	Reduces phosphorylation	Brewer et al. (2009)
700	691-700: PLTPSGEAPN	N → A	Abolishes phosphorylation	Brewer et al. (2009)
704	701-710: QALLRILKET	L → A	Abolishes phosphorylation	Brewer et al. (2009)
705	701-710: QALLRILKET	R → A	Abolishes phosphorylation	Brewer et al. (2009)
706	701-710: QALLRILKET	I → A	Abolishes phosphorylation	Brewer et al. (2009)
974	971-980: MARDPQRYLV	D → A	Strongly reduces phosphorylation	Jura et al. (2009)
977	971-980: MARDPQRYLV	R → A	Reduces phosphorylation	Jura et al. (2009)

Annotation: Red represents a mutant gene in the amino acid sequence.

### 3.2 Mechanism of EGFR-TKI therapy for NSCLC

EGFR is an important target protein for targeted therapy in cancer, especially non-small cell lung cancer. Abnormal changes, such as overexpression, enhanced activity and mutations of specific amino acid sites in the kinase domain, occur in a variety of tumor cells (Roskoski, 2014). Among non-small cell lung cancer patients, approximately 30%–40% of Asian patients have EGFR mutations (Herbst et al., 2008), approximately 90% of which are exon 19 deletion mutations (Del19) and exon 21 L858R mutations, accounting for 44%–51% and 38%–40% (Reita et al., 2021) of all mutations, respectively. The type of L858R EGFR mutant leads to destabilization of the nonactive conformation of EGFR so that it can spontaneously dimerize; this results in continuous activation of the kinase domain, which leads to continuous autophosphorylation of the C-terminal tyrosine site (Irmer et al., 2007; Reita et al., 2021). The type of Del19 EGFR mutant does not depend on dimerization (Jeonghee C et al., 2013).

With respect to the Del19 and L858R mutations, first-generation TKIs, such as gefitinib and erlotinib, can competitively bind to the ATP binding site of the EGFR tyrosine kinase domain, inhibit its kinase activity and autophosphorylation, and block EGFR-mediated signal transduction, thus inhibiting tumor cell proliferation (Pao and Chmielecki, 2010; Roskoski, 2014). However, most patients treated with first-generation TKI drug therapy develop mutations that cause acquired drug resistance, represented by EGFR T790M (exon 20) (49%–63%), after a progression-free survival period of approximately 10–14 months (Sequist et al., 2011; Tang and Lu, 2018). These mutations increase the affinity between EGFR and ATP while weakening the affinity of EGFR for TKIs, which leads to reactivation of the EGFR-mediated signal transduction pathway.

The third-generation TKI osimertinib is used mainly as a second-line therapy in patients with EGFR T790M mutations, but osimertinib can also be used as a first-line therapy in patients with Del19 and L858R mutations (Uchibori et al., 2018). However, the problem of acquired drug resistance will eventually occur. The representative types of mutations that cause drug resistance are T790M/C797S (during second-line therapy) and Del19/C797S and L858R/C797S (during first-line therapy). The mechanism of drug resistance is that C797 is a covalent binding site between osimertinib and EGFR, and a mutation at this site weakens the ability of osimertinib to bind to EGFR (Tang and Lu, 2018). To improve the targeted therapeutic effect of TKI drugs and prolong the progression-free survival time of patients, in addition to the development of a new generation of TKI drugs for drug-resistant mutations in EGFR, the exploration of a combination therapy strategy to increase the sensitivity of drug-resistant cell lines to TKI drugs is also considered a potentially effective approach (Yen et al., 2015).

### 3.3 Glycosylation characteristics of EGFR

**FIGURE 2:**
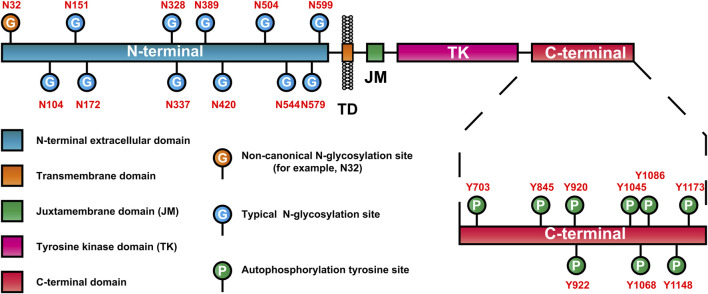
EGFR domain and distribution of glycosylation and autophosphorylation sites.

As mentioned earlier, EGFR is a highly glycosylated receptor tyrosine kinase, and the molecular weight of its glycosylated part (glycosylated chain) is approximately 40 kDa. At present, many glycosylation events at EGFR sites have been confirmed (Table 2). Moreover, EGFR consists of 11 typical N-glycosylation sites (NmurXMel Sstroke TMagre X is not proline), 4 atypical N-glycosylation sites (N-X-C) (Zhen et al., 2003; Takahashi et al., 2016) and a limited number of mucin-type O-glycosylation sites (Nguyen et al., 2009). These glycosylation sites are distributed mainly in the N-terminal extracellular domain of EGFR, as shown in Figure 2. In addition to the Asn337 site, which is a high-mannose-type sugar chain, the other glycosylation sites of EGFR are mainly sialylation- and (core) fucosylation-modified complex sugar chains, some of which also include isotypic branching structures and terminal fine structures, such as sLeX, sLeA, and LeY (Ferreira et al., 2018). The glycosylation characteristics of EGFR include glycosylation sites, the abundance distribution of glycosylation structures at each site (i.e., glycosylation microheterogeneity) and specific glycosylation types (e.g., sialylation and core fucosylation).

**TABLE 2:** Proven EGFR glycosylation sites.

EGFR modifications	Positions	Sequence status	Description	Publications
Glycosylation	56	51-60: LQRMFNNCEV	N-linked (GlcNAc.) (complex) asparagine; atypical; partial	Sato et al. (2000), Lu et al. (2010)
Glycosylation	73	71-80: QRNYDLSFLK	N-linked (GlcNAc.) asparagine; atypical	Lu et al. (2010)
Glycosylation	128	121-130: VLSNYDANKT	N-linked (GlcNAc.) asparagine	Zhen et al. (2003), Wu et al. (2005)
Glycosylation	175	171-180: DFLSNMSMDF	N-linked (GlcNAc.) asparagine	Lu et al. (2010)
Glycosylation	196	191-200: DPSCPNGSCW	N-linked (GlcNAc.) asparagine	Garrett et al. (2002)
Glycosylation	352	351-360: INATNIKHFK	N-linked (GlcNAc.) asparagine	Ferguson et al. (2003), Chen et al. (2009)
Glycosylation	361	361-370: NCTSISGDLH	N-linked (GlcNAc.) asparagine	Sato et al. (2000)
Glycosylation	413	411-420: PENRTDLHAF	N-linked (GlcNAc.) asparagine	Zhen et al. (2003)
Glycosylation	444	441-450: VSLNITSLGL	N-linked (GlcNAc.) asparagine	Garrett et al. (2002)
Glycosylation	528	521-530: RDCVSCRNVS	N-linked (GlcNAc.) asparagine	Mozzi et al. (2015)
Glycosylation	568	561-570: ECLPQAMNIT	N-linked (GlcNAc.) asparagine; partial	Sato et al. (2000)
Glycosylation	603	601-610: GENNTLVWKY	N-linked (GlcNAc.) asparagine; partial	Sato et al. (2000)
Glycosylation	623	621-630: HPNCTYGCTG	N-linked (GlcNAc.) (high mannose) asparagine	Zhen et al. (2003)

Annotation: Red indicates the glycosylation site in the amino acid sequence.

### 3.4 Crosstalk mechanism between the glycosylation and autophosphorylation of EGFR

Glycosylation has a fine regulatory effect on the biological function of EGFR through direct and indirect regulatory mechanisms (Ferreira et al., 2018). Among them, the indirect regulatory mechanism refers to the interaction between the GlcNAc terminus of the EGFR N-linked chain and ganglioside GM3, which inhibits signaling, whereas the interaction with GD3 enhances signaling (Milani et al., 2007; Liang et al., 2017). The direct regulatory mechanism means that glycosylation regulates the tyrosine kinase activity and autophosphorylation state of the EGFR protein by affecting its conformation, whereas the indirect regulatory mechanism means that EGFR glycosylation affects the turnover, metabolism and signal transduction of EGFR through its interaction with galectins or gangliosides. Under direct regulatory mechanisms, glycosylation characteristics, such as sialylation, core fucosylation, sugar chain branching and terminal fine structure, all have important effects on the autophosphorylation state of EGFR. These effects are generally believed to be achieved in two ways (Matsumoto et al., 2008):

• Glycosylation can change the binding affinity of EGFR for its ligands, thus regulating its kinase activity and autophosphorylation state. Sialylation of α2-3 and α2-6, loss of core fucose, a bispecific branching structure and a fine sLeX terminal structure decrease the affinity between EGFR and its ligands and decrease the level of casein autophosphorylation (Gu et al., 2004; Liu et al., 2011; Wang et al., 2015). Among these modifications, sialylation can even specifically regulate the phosphorylation level of specific tyrosine residues (such as Y1173) and thus shows site-specific regulation (Yen et al., 2015).
• Glycosylation can directly affect the ability of EGFR to form dimers, thus regulating its tyrosine kinase activity and autophosphorylation state in a ligand-independent manner (Fernandes et al., 2001). As previously mentioned, when EGFR contains specific site mutations (such as L858R), spontaneous dimerization occurs. Therefore, this process plays an important role in regulating kinase activity and autophosphorylation when EGFR is mutated. Studies have shown that the sugar chains at the Asn420 and Asn579 sites can inhibit the ligand-independent dimerization of EGFR and that the loss of sugar chains at the Asn579 site increases the conformational flexibility of EGFR, which leads to the dimerization of EGFR without ligand binding and increases its autophosphorylation level (Tsuda et al., 2000; Whitson et al., 2005).

**FIGURE 3:**
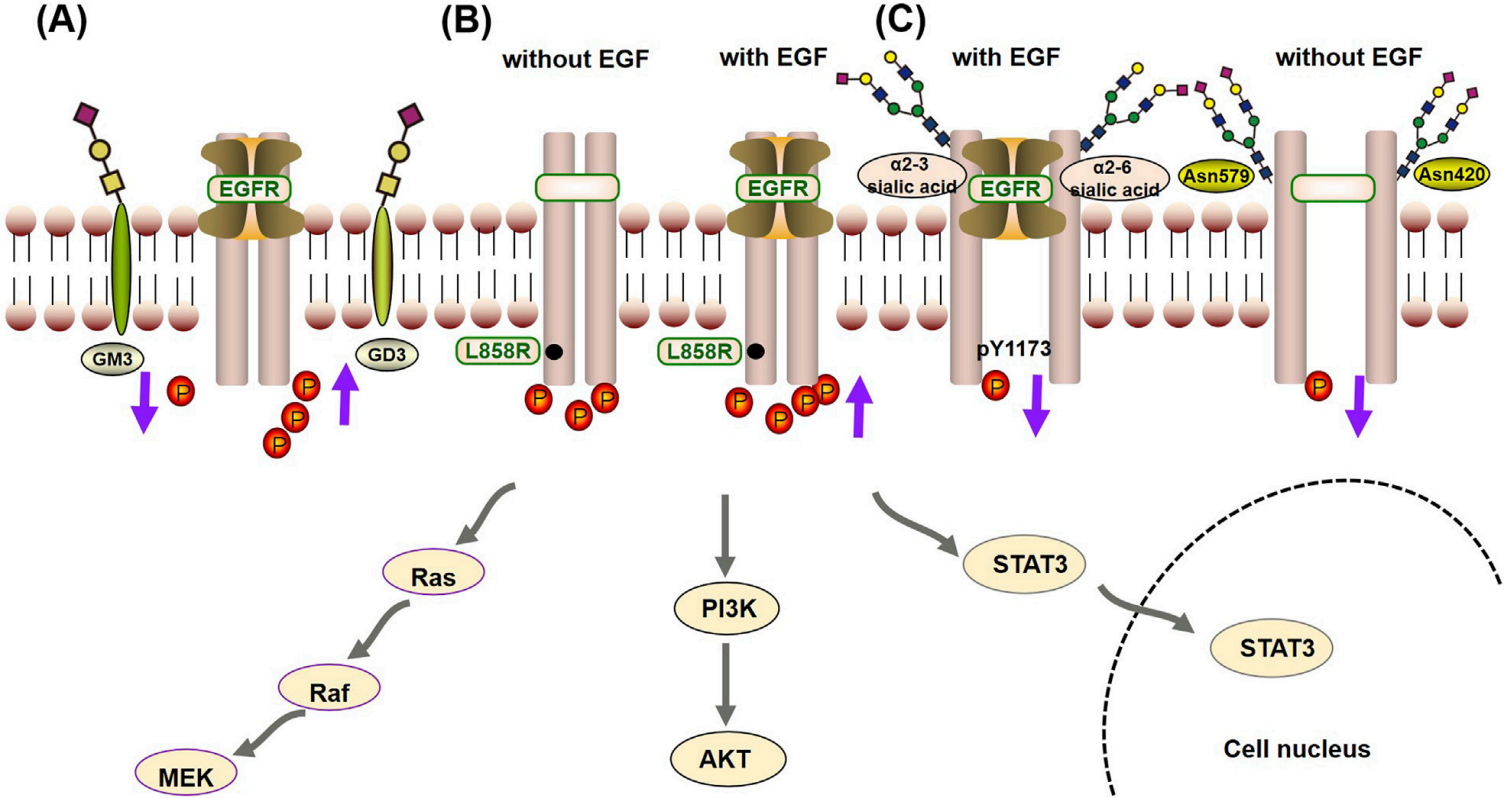
Crosstalk mechanism between glycosylation and autophosphorylation of EGFR: **(A)** The indirect regulatory mechanism, the interaction between the GlcNAc terminus of the EGFR N-linked chain and the ganglioside GM3, inhibits phosphorylation, thereby suppressing signal transduction. On the other hand, interaction with GD3 promotes phosphorylation, enhancing signaling. **(B)** When EGFR contains specific site mutations (such as L858R), it promotes its kinase activity and autophosphorylation state independently of EGF, but it is also enhanced by EGF. **(C)** In the direct regulatory mechanism, glycosylation can change the binding affinity of EGFR for its ligands (such as α2-3 and α2-6 sialic acid), reducing the occurrence of phosphorylation. Furthermore, sialylation can specifically inhibit the phosphorylation level of specific tyrosine residues (such as Y1173). At last, the sugar chains at the Asn420 and Asn579 sites can inhibit the ligand-independent dimerization of EGFR, reducing the autophosphorylation level.

A fine regulatory relationship between the glycosylation characteristics of EGFR and its autophosphorylation state has been described, and it is worth noting that the direct regulatory mechanism of glycosylation (especially through the second pathway) also has an important effect on drug sensitivity in TKI drug resistance, as shown in Figure 3. The deglycosylation, sialylation and core fucosylation of EGFR can affect its autophosphorylation state and the sensitivity of EGFR-TKI-resistant mutant cells (L858R/T790M and Del19/T790M) to gefitinib or erlotinib (Matsumoto et al., 2008; Ling et al., 2009; Park et al., 2012; Britain et al., 2018). Since the glycosylation of EGFR can affect the sensitivity of drug-resistant cells to TKIs by regulating the autophosphorylation state of EGFR and because the glycosylation characteristics of EGFR are regulated mainly by glycosyltransferases and glycosidases in the Golgi apparatus and endoplasmic reticulum, glycosyltransferases or glycosidases have become potential effective targets for enhancing sensitivity to TKIs (Lopez Sambrooks et al., 2018).

## 4 Conclusion

Proteins are the executors of all cellular activities and embody biological functions. The existence of PTMs greatly increases the complexity of proteins. Studying the PTMs of proteins is crucial for understanding the mechanism of intracellular signal transduction, elucidating the physiological functions of life, and determining the pathogenic mechanism of related diseases. The study of PTMs can help us identify additional potential drug targets. In recent years, with the rapid development of proteomics technology based on biological mass spectrometry, especially for phosphorylation, the identification of PTM sites has tended to be saturated, and the study of PTMs has gradually shifted from qualitative to quantitative studies. Combined with this exquisite experimental design, the dynamic changes in PTMs over time and at different locations have been characterized, and finally, the dynamic changes in PTMs have been shown to be related to their biological function. With the further study of PTMs, crosstalk between PTMs has gradually become an urgent problem.

The primary premise of target discovery in EGFR-TKI therapy is to explore the key glycosylation characteristics related to TKI drug sensitivity. On this basis, the correlation between the key glycosylation characteristics and the autophosphorylation state of EGFR can be further established, which can lay a foundation for the systematic interpretation of the scientific principle by which EGFR glycosylation regulates the sensitivity of drug-resistant mutants to TKIs (Yen et al., 2015). Currently, the correlations between EGFR glycosylation characteristics and the autophosphorylation state of EGFR, as well as the effect of glycosylation on TKI drug sensitivity, are still largely limited to first-generation TKI drugs, and systematic studies on third-generation TKI drugs, such as osimertinib, are scarce. Therefore, further clinical sample analysis is needed to identify and verify the key differential glycosylation characteristics, establish correlations between those characteristics and the EGFR autophosphorylation state, identify therapeutic targets that affect the sensitivity of the third-generation TKI drug osimertinib, and provide key information on glycosylation characteristics.
